# Climatic Niche Dynamics and Potential Distribution of the Invasive Sweet Potato Weevil (*Cylas formicarius*) in China

**DOI:** 10.3390/biology15100785

**Published:** 2026-05-15

**Authors:** Yuxi Wang, Min Liu, Yaqian Shang, Hina Gul, Chuanlin Yin, Shuxing Zhou, Chizhou Liang, Jianzhong Li, Jinming Zhang

**Affiliations:** 1 State Key Laboratory for Quality and Safety of Agro-Products, Key Laboratory of Biotechnology in Plant Protection of Ministry of Agriculture (MOA) of China and Zhejiang Province, Institute of Plant Protection and Microbiology, Zhejiang Academy of Agricultural Sciences, Hangzhou 310021, China; wangyuxi0027@163.com (Y.W.); liumin_198110@163.com (M.L.); gulhina680@gmail.com (H.G.); 3090100232@zju.edu.cn (S.Z.); 2 College of Life and Environmental Sciences, Hangzhou Normal University, Hangzhou 311121, China; 13323871140@163.com; 3 Department of Biology, College of Life Science, China Jiliang University, Hangzhou 310018, China; chuanlinyin@cjlu.edu.cn; 4 Zhejiang Provincial Plant Protection, Quarantine and Pesticide Management Station, Hangzhou 310020, China; czliang1975@163.com; 5 Center of Agro-Technology and Extension of Qujiang, Quzhou 324002, China

**Keywords:** Biomod2, climatic niche dynamics, *Cylas formicarius*, ensemble forecasting, invasion risk, quarantine surveillance

## Abstract

To assess the potential risk of expansion of the sweet potato weevil (*Cylas formicarius*) in China under climate change, we combined principal component analysis in environmental space (PCA-env) with a Biomod2 ensemble model, using 173 occurrence records from its native range in India and its invaded range in China. We quantified the dynamics of the climatic niche between the native and invaded ranges and projected both current and future climatically suitable areas in China. Precipitation during the wettest month (Bio13), mean temperature during the driest quarter (Bio9), and isothermality (Bio3) were the key climatic predictors. Niche overlap between India and China was low (Schoener’s *D* = 0.107). The invaded niche was characterized by high stability (0.991) with very limited expansion (0.009), indicating strong niche conservatism. However, a relatively high unfilling value (0.633) suggests that the species has not yet occupied all potentially suitable climatic space in China. The current suitable area was estimated at 37.55 × 10^4^ km^2^, primarily concentrated in South China and the southeastern coastal region. Under future climate scenarios, suitable habitat is projected to expand overall, extending into Central, Eastern, and Southwestern China. This study provides a climate-informed forecasting framework for assessing the potential spread of *C. formicarius* in China and offers practical support for quarantine surveillance and region-specific management.

## Simple Summary

The sweet potato weevil (*Cylas formicarius*) is one of the most damaging pests of sweet potato and can seriously reduce both yield and quality. In China, this pest is currently found mainly in southern regions, but rising temperatures may allow it to spread further. In this study, we examined how the pest responds to climate conditions and predicted where it could occur in China now and in the future. We found that the weevil has largely kept the same climate preferences in China as in its native range, but it has not yet spread to all the areas that are already suitable for it. At present, the most favorable areas are mainly in South China and along the southeastern coast. Under future climate change, these suitable areas are expected to expand into Central, Eastern, and Southwestern China. This means the pest may continue to spread and threaten more sweet potato-growing regions. Our findings provide useful guidance for pest surveillance, quarantine, and early management in areas that may face future invasion risk.

## 1. Introduction

Biological invasions have emerged as one of the primary ecological threats to sustainable agriculture and are being exacerbated by ongoing climate change. Climate warming, more frequent extreme events, and the intensification of international trade have significantly increased the likelihood that alien species will spread across regions and establish new populations [1,2,3]. Previous studies have demonstrated that, under climate change, the potential distributions of many agricultural pests are shifting toward higher latitudes and elevations [4], thereby posing persistent threats to agricultural production and food security. Since prevention remains the most cost-effective strategy for combating alien invasions, accurately identifying regions at risk of invasion is crucial for developing effective surveillance and management programs.

Climate is a critical environmental factor that shapes species distributions and plays a central role in regulating the spread, establishment, and outbreak dynamics of invasive species [5]. Niche theory posits that the geographic distribution of a species is largely constrained by its climatic niche [6]. Ecological niche models (ENMs) are extensively used to estimate the potential suitable habitat of species by linking occurrence records to environmental variables, and they have become essential tools for assessing invasion risk [7]. In the context of invasive species, niche conservatism is often a key assumption when using ENMs [8,9]. However, invasive species may encounter different environmental conditions in their introduced ranges, leading to potential niche shifts under novel selective pressures [10,11]. PCA-env-based climatic niche analysis offers a robust method for quantifying overlap, stability, expansion, and unfilling between native and invaded ranges, providing a direct way to evaluate niche dynamics [9,12,13].

The sweet potato weevil (*Cylas formicarius*) is the most destructive insect pest of sweet potato and a significant quarantine pest in sweet potato-growing regions worldwide [14]. Both adults and larvae damage vines, petioles, leaf veins, and storage roots, leading to reductions in both yield and quality during cultivation and storage [15]. Native to the Indian subcontinent, *C. formicarius* spread across Asia via the movement of sweet potato planting materials and was introduced to China in the nineteenth century [16]. It is currently concentrated in southern China, but recent warming has shifted its suitable range northward, and substantial damage is now extending into sweet potato production areas along the Yangtze River basin [17]. As such, accurately predicting where the climate is suitable for the species is important for early detection and targeted management.

Species distribution models (SDMs) are widely employed to assess invasion risk and forecast potential distributions under environmental change [18]. Machine learning approaches, including random forest, maximum entropy, and boosted regression tree models, have been extensively applied in studies of invasive species and pest distributions [19]. However, predictions from individual algorithms can vary significantly because model structure and assumptions about response forms influence extrapolation to novel environments [19,20]. Ensemble forecasting helps mitigate this uncertainty by integrating predictions from multiple models [21]. In R 4.4.3, biomod2 offers a standardized framework for fitting a range of statistical and machine learning SDMs and generating weighted ensemble predictions based on model evaluation scores [22]. In this study, we used occurrence records from the native range (India) and invaded range (China) of *C. formicarius* to (i) quantify climatic niche dynamics in invaded China using PCA-env and (ii) predict the current and future suitable areas of the species in China using the Biomod2 ensemble model. Unlike distribution-only assessments, this study first quantified whether the climatic niche of *C. formicarius* has shifted during invasion and then used this niche dynamics evidence to support ensemble projections under current and future climates. By linking PCA-env-derived niche stability, expansion, and unfilling with Biomod2-based suitability forecasts, the study distinguishes currently occupied areas, climatically suitable but unfilled areas, and future expansion fronts, thereby providing a more informative framework for quarantine surveillance and regional pest management.

## 2. Materials and Methods

### 2.1. Occurrence Data

**Figure 1:**
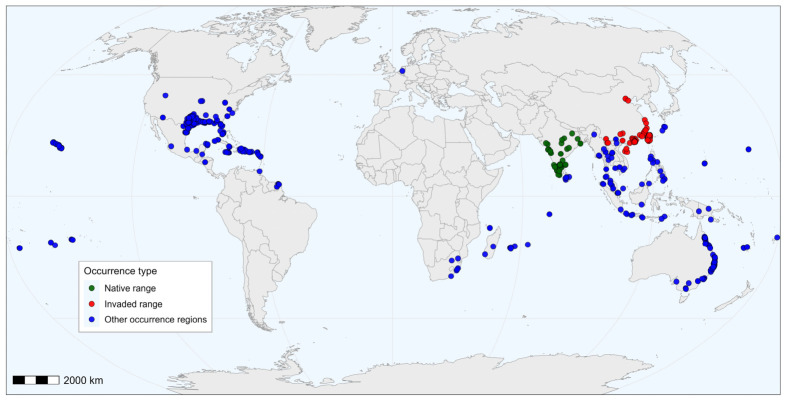
Records of native and invaded occurrences of *Cylas formicarius* worldwide. Green points indicate native-range records from India, red points indicate invaded-range records from China, and blue points indicate records from other occurrence regions. The base map was obtained from the Standard Map Service of the Ministry of Natural Resources of the People’s Republic of China, with map approval number GS(2016)1560. The map is used only for scientific illustration, and the boundary representation does not imply any political or administrative position of the authors or publisher.

Occurrence records were compiled from field surveys, the Global Biodiversity Information Facility (GBIF, https://www.gbif.org/ (accessed on 23 January 2026)) [23], and published literature. A total of 183 global occurrence records were initially collected. Before spatial thinning, occurrence records were checked for geographic reliability. Duplicate coordinates, records lacking coordinates, records with uncertain or low-precision locality information, records falling outside the reported country or province, and records located in water bodies were removed. To reduce sampling bias and spatial autocorrelation, all records were spatially filtered before modeling. For the invaded range in China, a 5 km thinning distance was used because the 2.5 arc-min bioclimatic layers correspond approximately to a 4.5–5 km grid size across much of the study area; this distance therefore reduced spatial clustering and the likelihood that multiple records represented the same or adjacent climate pixels while retaining sufficient occurrence data for model calibration. Records from the invaded range were thinned with the spThin package (version 0.2.0) in R (version 4.4.3) [24], using a minimum nearest-neighbor distance of 5 km with one random repetition. Native-range records from India were not thinned because of their limited sample size. After thinning, 173 records were retained for subsequent analyses (Figure 1), including 127 records from China (invaded range) and 46 from India (native range).

### 2.2. Environmental Variable Selection

All 19 bioclimatic variables at 2.5 arc-min resolution were downloaded from WorldClim v2.1 and used as candidate predictors for niche analyses and species distribution modeling [25]. Current climate data represented the period 1970–2000. Future bioclimatic variables were obtained from the WorldClim v2.1 CMIP6 downscaled climate database at 2.5 arc-min resolution. Projections were based on the BCC-CSM2-MR global climate model, under four shared socioeconomic pathways (SSP126, SSP245, SSP370, and SSP585) for two future periods: 2041–2060 (2050s) and 2081–2100 (2090s). The same climate model source was used consistently across all scenarios and periods [26].

To avoid overfitting caused by multicollinearity, a combined SDM- and statistics-based screening procedure was used [27]. First, a Maxnet model was trained on the occurrence data, and variable importance was assessed using permutation importance. Only variables with importance > 5% were retained. Pearson correlation coefficients were then calculated among candidate variables using environmental background data, and variance inflation factors (VIFs) were further used for screening. Variables with pairwise |r| > 0.8 or VIF > 10 were removed sequentially [28]. Three key climatic variables were finally retained for niche analyses, and their contributions to the first two PCA-env axes are presented in Table 1.

### 2.3. Climatic Niche Dynamics and Overlap Analysis

Climatic niche dynamics were quantified within the COUE framework proposed by Broennimann et al. [12]. Occurrence records from the native range (India) and invaded range (China), together with the selected climatic variables, were projected onto the first two PCA-env axes using a 100 × 100 grid [8]. Kernel density smoothing was used to estimate occurrence density in environmental space and generate smoothed niche occupancy surfaces [13]. Niche dynamics were partitioned into stability (overlap between native and invaded niches), expansion (environmental space occupied only in the invaded range), and unfilling (environmental space occupied in the native range but not in the invaded range) [29].

Niche overlap was quantified using Schoener’s *D*, which ranges from 0 (no overlap) to 1 (complete overlap) [12]. Niche equivalency and similarity tests were further performed to assess the significance of niche differences. In the equivalency test, occurrence records from the native and invaded ranges were randomly reassigned to generate a null distribution of *D* values. In the similarity test, occurrence density in one range was randomly shifted within the available background environment and compared with the niche occupied in the other range [30]. Each test was repeated 1000 times [31]. Analyses were conducted with the ecospat package (version 4.1.3) in R (version 4.4.3) [13].

### 2.4. Ensemble Species Distribution Modeling

Potential distribution was modeled with the biomod2 package (version 4.3) in R (version 4.4.3) using ensemble forecasting to reduce uncertainty from single algorithms [22,32]. Ten algorithms were considered: generalized linear models (GLMs), generalized boosted regression models (GBMs), generalized additive models (GAMs), classification tree analysis (CTA), artificial neural networks (ANNs), flexible discriminant analysis (FDA), surface range envelope (SRE), multivariate adaptive regression splines (MARS), random forest (RF), and maximum entropy (MaxEnt). For MaxEnt, background data were generated automatically from environmental rasters; the other models were run with default settings. Pseudo-absence data were generated by random sampling from the study area background, with 2000 pseudo-absences per run and three repetitions. Occurrence and pseudo-absence points were given equal weights to balance the samples. Data were split randomly into training and testing subsets at a 7:3 ratio. Each model was repeated four times for each pseudo-absence set, yielding 120 model runs in total. Model performance was evaluated using the area under the receiver operating characteristic curve (AUC) and the true skill statistic (TSS). After cross-validation, only models with valid evaluation statistics and TSS > 0.80 were retained for ensemble forecasting. Ensemble weights were calculated from the TSS values of the retained models as wi = TSSi/sum(TSSi) so that better-performing algorithms contributed more strongly to the final prediction.

### 2.5. Habitat Classification and Spatial Analysis

Continuous habitat-suitability probabilities (P) from the ensemble model were used to classify the potential distribution of *C. formicarius*. The study area was divided into five classes of habitat suitability based on the following probability ranges: unsuitable (0 ≤ P < 0.2), low suitability (0.2 ≤ P < 0.4), moderate suitability (0.4 ≤ P < 0.6), high suitability (0.6 ≤ P < 0.8), and very high suitability (0.8 ≤ P < 1.0) [33]. The optimal ROC threshold was determined by maximizing the sum of sensitivity and specificity, equivalent to maximizing TSS (sensitivity + specificity − 1), and was used to convert continuous suitability predictions into binary suitable/unsuitable maps. Spatial processing, area calculations, and map production were carried out in ArcGIS Pro (version 3.6; Esri, Redlands, CA, USA). Areas shifting from suitable to unsuitable were defined as loss, areas shifting from unsuitable to suitable as gain, and areas remaining suitable under both scenarios as unchanged. Spatial dynamics of suitable habitat were quantified by comparing these area types.

## 3. Results

### 3.1. Climatic Niche Dynamics

**Figure 2:**
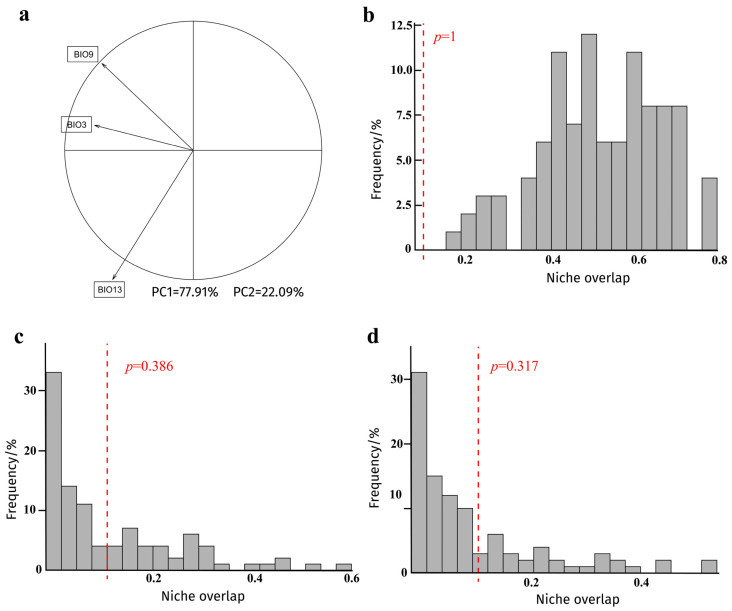
Principal component analysis and niche test results for *Cylas formicarius*: (**a**) contributions of environmental variables to the principal component axes; (**b**) niche equivalency test of niche overlap between the native range (India) and the invaded range (China); (**c**,**d**) representative histograms of niche similarity tests. Gray bars indicate simulated values, and red vertical lines indicate observed values. *p* values represent the probability that the simulated *D* value is greater than or equal to the observed *D* value under randomization.

PC1 and PC2 explained 77.91% and 22.09% of the environmental variation, respectively. PC1 represented the main composite gradient of temperature and precipitation variables and therefore captured the dominant climatic constraints on the niche of *C. formicarius* (Figure 2a).

Niche overlap between the native range (India) and the invaded range (China) was low (Schoener’s *D* = 0.107; Hellinger’s *I* = 0.298). Neither the niche equivalency test (Figure 2b) nor the niche similarity tests (Figure 2c,d) was significant (*p* > 0.05), indicating that the climatic niches occupied in China and India were not statistically equivalent and that the observed overlap was not significantly greater than expected under random conditions.

**Figure 3:**
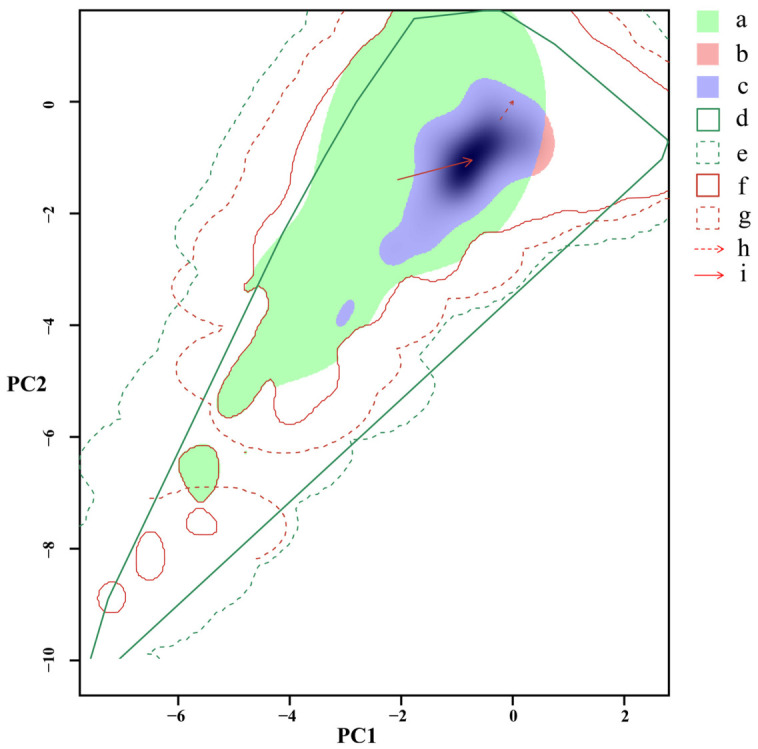
Comparison of the climate niches between the populations of *Cylas formicarius* in its native (India) and invasive (China) habitats: (a) climate niche of the native population (India); (b) climate niche of the invasive population (China); (c) niche overlap between the native (India) and invasive (China) populations; (d) environmental niche available to the native populations (India) with 100%; (e) environmental niche available to the native populations (India) with 50%; (f) environmental niche available to the invasive populations (China) with 100%; (g) environmental niche available to the invasive populations (China) with 50%; (h) background environmental range shifted from the native range (India) to the invasive range (China); (i) centroid of climate niche shifted from the native range (India) to the invasive range (China). PC: principal component.

Niche dynamics analysis showed that the invaded niche was dominated by stability (0.991), with very limited expansion (0.009) but substantial unfilling (0.633) (Figure 3). Both niche centroid shift and background environmental shift were detected. Overall, the climatic niche occupied in China was largely a subset of that in India, indicating strong niche conservatism.

### 3.2. Model Evaluation and Current Climatic Suitability

Model performance was evaluated using the combined ROC-TSS criteria. Of the 10 candidate algorithms, eight yielded valid evaluation results; FDA and MaxEnt did not produce stable or effective validation metrics during cross-validation and were excluded from the accuracy comparison. CTA and MARS showed the best overall performance, followed by GBM, GAM, and GLM, whereas RF and SRE performed less well. The ensemble model constructed from high-performing single models (TSS > 0.80) further improved prediction accuracy, with AUC = 0.961 and TSS = 0.829, exceeding the performance of all single models, including the best CTA model. Subsequent analyses were therefore based on the ensemble model.

**Figure 4:**
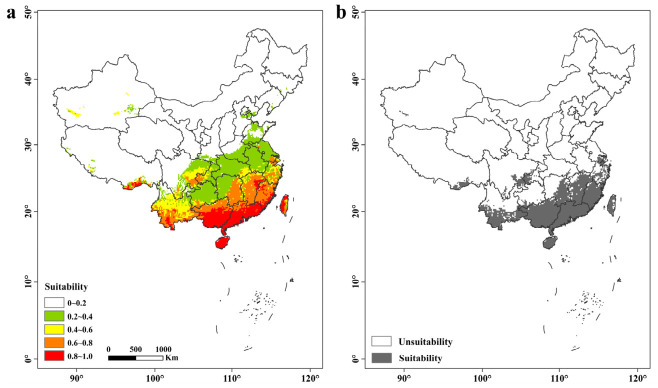
Potential distribution map in the current period (**a**) and binary distribution map (**b**) of *Cylas formicarius* based on combinatorial models. This figure was prepared based on the standard map with approval number GS(2020)4619, obtained from the Standard Map Service of the Ministry of Natural Resources of the People’s Republic of China. The base map was not modified. The map is used only for scientific illustration, and the boundary representation does not imply any political or administrative position of the authors or publisher.

The current potential distribution of *C. formicarius* in China showed a clear south-to-north decline in suitability (Figure 4a). Very high suitability (0.8 ≤ P < 1.0) and high suitability (0.6 ≤ P < 0.8) were concentrated mainly in South China and the southeastern coastal region, including Guangdong, Guangxi, Hainan, Taiwan, and southern Fujian, forming contiguous high-risk areas. Moderately suitable areas (0.4 ≤ P < 0.6) were distributed mainly across the middle and lower Yangtze River basin and Southwest China, forming a transition belt from south to north. Low-suitability areas (0.2 ≤ P < 0.4) occurred sporadically in southern North China and marginal parts of Southwest China. In contrast, the arid northwest, the Qinghai–Tibet Plateau, and most of Northeast China were unsuitable (0 ≤ P < 0.2). Overall, the predicted climatically suitable range was concentrated in warm and humid regions of southern China. The binary map estimated a total suitable area of 37.55 × 10^4^ km^2^ (Figure 4b).

### 3.3. Future Climatically Suitable Areas in China

**Figure 5:**
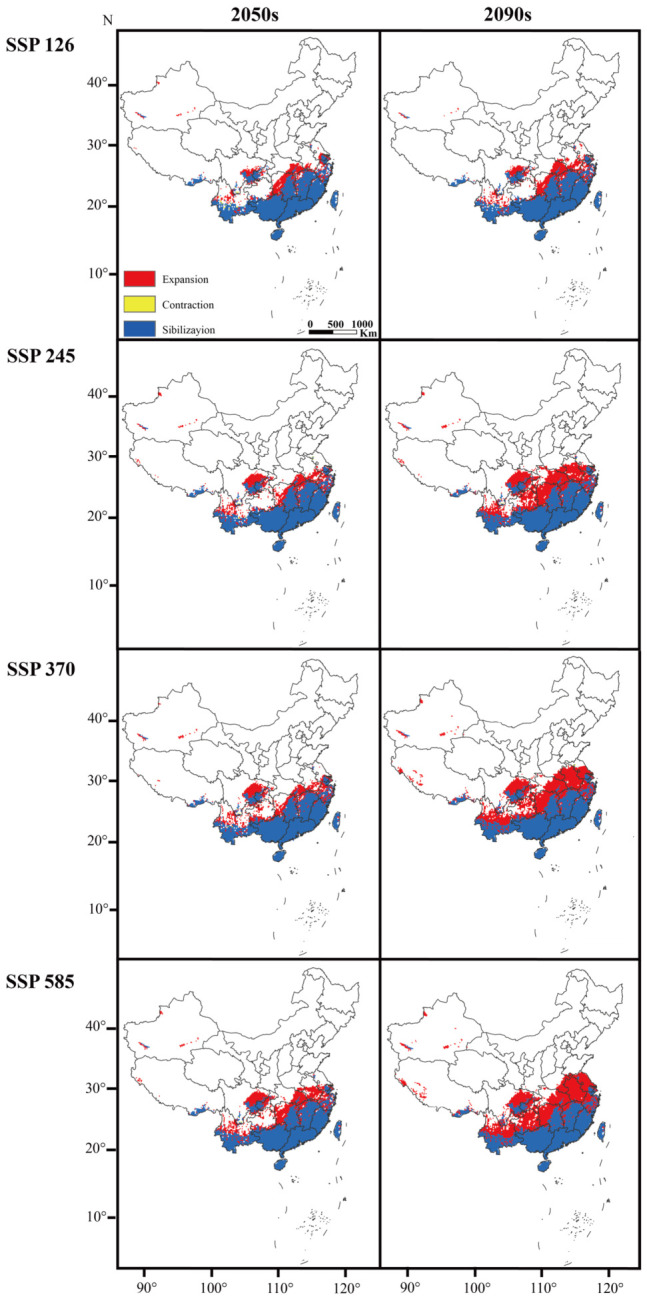
Spatial distribution pattern of *Cylas formicarius* under different climate scenarios. 2050s: 2041–2060; 2090s: 2081–2100. This figure was prepared based on the standard map with approval number GS(2020)4619, obtained from the Standard Map Service of the Ministry of Natural Resources of the People’s Republic of China. The base map was not modified. The map is used only for scientific illustration, and the boundary representation does not imply any political or administrative position of the authors or publisher.

Under all future climate scenarios, climatically suitable habitat expanded markedly, and the magnitude of expansion increased with emission intensity and time horizon (Table 2). Under SSP126, the suitable area increased to 42.19 × 10^4^ km^2^ in the 2050s and 45.05 × 10^4^ km^2^ in the 2090s. Newly suitable areas were mainly projected into Hunan, southern Jiangxi, and the southern margin of the Sichuan Basin. Under SSP245 and SSP370, suitable area reached 57.60 × 10^4^ km^2^ and 61.24 × 10^4^ km^2^, respectively, by the 2090s, and the northern suitability boundary shifted clearly into the middle-lower Yangtze River basin and the Jianghuai region, with stronger connectivity across Central and East China. The greatest expansion occurred under SSP585, where the suitable area reached 65.27 × 10^4^ km^2^ by the 2090s, representing a 101.94% increase over the current period. Across all scenarios, loss areas were small (loss ratio ≤ 0.72%), unchanged areas remained close to 32 × 10^4^ km^2^, and the overall pattern was a persistent northward expansion from South China toward Central and East China, with extension into Southwest China (Figure 5).

## 4. Discussion

The current core suitable areas of *C. formicarius* are concentrated in the warm and humid regions of South China and the southeastern coast. Under all future scenarios, suitability expands northward, with the extent of expansion increasing as warming intensity and time progress. This pattern aligns with the general trend for agricultural pests to shift toward higher latitudes and elevations under global warming [34,35]. Expansion was modest under SSP126 and most pronounced under SSP585, under which the suitable area more than doubled by the 2090s. Meanwhile, retained habitat remained stable, and contraction was minimal across scenarios, suggesting that South China and the southeastern coast will remain long-term core suitable regions. In contrast, Central China, East China, and transitional zones in Southwest China will become increasingly important risk frontiers. The model also identified several isolated suitable patches in Xinjiang. Since only climatic variables were considered here, and factors such as host availability, irrigation, topographic barriers, and dispersal processes were not incorporated, these patches should be interpreted as climatically suitable rather than confirmed invasion destinations [18,36]. From a management perspective, these projections are particularly valuable for risk zoning, the forward deployment of surveillance resources, and precision interventions, rather than broad, calendar-based pesticide applications [37].

Projecting future suitable areas with SDMs implicitly assumes that the climatic niche of the species is at least partly conserved through time and space. Our PCA-env results provide support for this assumption because the invaded niche in China showed very high stability and minimal expansion relative to the native niche. Therefore, the future suitability maps should be interpreted as climate-based forecasts under niche conservatism, with the high unfilling value indicating that unoccupied but climatically suitable regions in China may represent potential future invasion fronts. Alternative assumptions, such as rapid climatic niche evolution or adaptation to novel environments, could not be directly tested with occurrence-only data and should be evaluated in future studies using physiological experiments, population-level data, or mechanistic models.

Changes in climatic niche characteristics are crucial for determining whether an invasive pest has adapted to its new range [9,38]. While low niche overlap alone indicates differences in the environmental space occupied by the species in its native and invaded ranges, it does not, by itself, demonstrate significant niche expansion [31]. In this study, niche overlap between India and China was low (*D* = 0.107), while stability was exceptionally high (0.991) and expansion was minimal (0.009). These results suggest that the climatic space already occupied in China largely remains within the ancestral niche envelope, indicating that *C. formicarius* still exhibits strong niche conservatism [8,9]. This pattern aligns with the observation that many invasive species largely retain their original climatic niche after introduction [8,31]. Therefore, the spread of *C. formicarius* in China is more likely to reflect the continued filling of pre-existing suitable climatic space rather than the rapid evolution of a novel climatic niche [39].

At the same time, the high unfilling value (0.633) suggests that the current realized distribution of *C. formicarius* in China has not yet equilibrated with the full extent of climatically suitable habitat [8,9]. The species is readily transported through infested vines, storage roots, and planting materials while also retaining some natural dispersal capacity [40,41]. As a result, its known distribution may underestimate its actual invasion potential [42]. In areas already predicted to be suitable, continued movement of infested seed roots, seedlings, or marketable tubers under inadequate quarantine measures could further occupy currently unfilled but suitable environmental space [43,44]. From the perspective of niche dynamics, *C. formicarius* appears to remain in an active expansion phase in China. As a cryptic pest that targets underground sweet potato storage roots, the successful establishment and spread of *C. formicarius* within suitable areas depend not only on climatic suitability [17,45] but also on factors such as host continuity, cropping systems, and human-mediated transport. The current core suitable areas, future expansion fronts, and marginal risk zones predicted in this study can guide targeted quarantine, transport inspection, trace-back verification, and differentiated surveillance [46]. A tiered management strategy is therefore recommended: integrated source reduction, field sanitation, and ecologically based suppression in core high-suitability areas; intensified monitoring, pheromone-based surveillance, movement control, and rapid localized eradication of new outbreaks at expansion fronts; and routine surveillance, transport inspection, and emergency preparedness in marginal low-suitability areas [37,44,47]. Within this framework, chemical insecticides should be considered primarily as selective and targeted emergency tools for outbreak or introduction scenarios, rather than the default approach for long-term management.

## 5. Conclusions

By integrating PCA-env-based climatic niche dynamics with a Biomod2 ensemble model, this study assessed the climatic niche characteristics and potential suitable distribution of *C. formicarius* in China. Precipitation of the wettest month, mean temperature of the driest quarter, and isothermality were identified as the key climatic drivers. The species exhibited strong niche conservatism in China, but the high unfilling value suggests considerable potential for further spread. Current suitable areas are concentrated in South China and the southeastern coastal region, while future suitable areas expand under all four SSP scenarios, with the main expansion front extending into Central China, East China, and transitional zones in Southwest China. Overall, *C. formicarius* remains at risk of continued expansion and northward spread in China. These findings support the development of climate-informed, region-specific strategies for quarantine regulation, surveillance, early warning, and coordinated pest management.

## Figures and Tables

**Table 1:** Environmental variables in the climate niche shift analysis of *Cylas formicarius*.

Variable	Code	Unit	PC1 Contribution (%)	PC2 Contribution (%)	Total Contribution (%)
Precipitation of wettest month	BIO13	mm	20.59	14.82	35.42
Mean temperature of driest quarter	BIO9	°C	26.44	6.71	33.15
Isothermality	BIO3	%	30.87	0.56	31.43

**Table 2:** Spatial distribution pattern of *Cylas formicarius* under different climate scenarios.

Climate Scenario	Period	Total Suitable Area (×10^4^ km^2^)	Loss Area (×10^4^ km^2^)	Gain Area (×10^4^ km^2^)	Unchanged Area (×10^4^ km^2^)	Loss Ratio (%)	Gain Ratio (%)	Overall Change (%)
Current		37.55						
SSP126	2050s	42.19	0.23	10.11	32.09	0.72	31.28	30.55
	2090s	45.05	0.07	12.80	32.25	0.23	39.61	39.38
SSP245	2050s	45.40	0.11	13.20	32.21	0.34	40.83	40.49
	2090s	57.60	0.05	25.33	32.27	0.15	78.39	78.23
SSP370	2050s	46.60	0.07	14.35	32.25	0.23	44.41	44.18
	2090s	61.24	0.04	28.96	32.28	0.11	89.61	89.50
SSP585	2050s	49.97	0.05	17.70	32.27	0.15	54.76	54.60
	2090s	65.27	0.00	32.95	32.32	0.00	101.94	101.94

2050s: 2041–2060; 2090s: 2081–2100.

## Data Availability

The occurrence records used in this study were compiled from field surveys, GBIF, and published literature. Environmental data are publicly available from WorldClim. The processed occurrence dataset and model outputs are available from the corresponding author upon reasonable request.
